# RETRACTION: Novel Synthesis of Titanium Oxide Nanoparticles: Biological Activity and Acute Toxicity Study

**DOI:** 10.1155/bca/9854987

**Published:** 2026-04-08

**Authors:** Bioinorganic Chemistry and Applications

RETRACTION: M. T. Hamed, B. A. Bakr, Y. H. Shahin, et al., “Novel Synthesis of Titanium Oxide Nanoparticles: Biological Activity and Acute Toxicity Study,” *Bioinorganic Chemistry and Applications*, 2021, 8171786, https://doi.org/10.1155/2021/8171786.

The above article, published online on 12 August 2021 in Wiley Online Library (https://wileyonlinelibrary.com), has been retracted by agreement the handling Editor, Guillermo Mendoza‐Diaz, and John Wiley & Sons Ltd. The retraction has been agreed due to multiple concerns identified with the figures. The authors initially contacted the publisher to correct errors in Figure 3, which had also been raised on PubPeer [1]. During the course of the investigation, additional concerns were identified by the publisher in Figures 4 and 5. The concerns were presented to the authors with a request for original raw data and an explanation. The authors further requested a correction to Figure 10c. A summary of the concerns is below:

• In Figure 3, the graphs are duplicates
• In Figure 4, the untreated control panels:
  ◦ are identical to the untreated control panels in Figure 3b of [2].
  ◦ are highly similar to the untreated control panels in Figure 3 of [3].
• In Figure 4, the untreated control/Caco‐2 and untreated control/MDA‐MB 231 panels are identical to the untreated control/Caco‐2 and untreated/MDA‐MB 231 panels in Figure 5b of [4].
• In Figure 4, the untreated control/Caco‐2 and the control/HepG‐2 panels are similar to the control/Caco‐2 and the control/HepG‐2 panels in Figure 10 of [5].
• In Figure 4, the untreated control/Caco‐2 and the control/HepG‐2 panels are similar to the control/Caco‐2 and the control/HepG‐2 panels in Figure 8 of [6].
• In Figure 4, the untreated control/Caco‐2 panel is identical to the untreated control/Caco‐2 panel in Figure 2 of [7].
• In Figure 5, panel a is identical to the kidney A panel in Figure 5 of [2].
• In Figure 10, the authors confirmed that the incorrect image was selected during figure preparation

After assessment of the concerns and the author’s response by the editorial board, the results and conclusions are no longer deemed reliable, and the article is retracted.

The authors disagree with the retraction.
